# Rheological Analysis of Binary Eutectic Mixture of Sodium and Potassium Nitrate and the Effect of Low Concentration CuO Nanoparticle Addition to Its Viscosity

**DOI:** 10.3390/ma8085194

**Published:** 2015-08-11

**Authors:** Mathieu Lasfargues, Hui Cao, Qiao Geng, Yulong Ding

**Affiliations:** 1 Institute of Particle Science and Engineering, University of Leeds, Leeds LS2 9JT, UK; E-Mail: m.lasfargues@leeds.ac.uk; 2 School of Chemical Engineering, University of Birmingham, Birmingham B15 2TT, UK; E-Mails: h.cao@bham.ac.uk (H.C.); GXQ471@student.bham.ac.uk (Q.G.)

**Keywords:** rheology, molten salt, solar salt, nanoparticle, copper oxide, viscometer

## Abstract

This paper is focused on the characterisation and demonstration of Newtonian behaviour of salt at both high and low shear rate for sodium and potassium nitrate eutectic mixture (60/40) ranging from 250 °C to 500 °C. Analysis of published and experimental data was carried out to correlate all the numbers into one meaningful 4th order polynomial equation. Addition of a low amount of copper oxide nanoparticles to the mixture increased viscosity of 5.0%–18.0% compared to the latter equation.

## 1. Introduction

One of the first papers to investigate the rheology of molten salt was carried out by Janz *et al*., using oscillating sphere technique [1]. Whilst the publication displayed large amounts of accumulated data for various salts, it did not provide a description of the setup used and the uncertainty in the measurements gathered was given to plus or minus ten percent. This was not the case with Nissen which provided a detailed description of the apparatus used to determine viscosity via the principle of dampening through the use of one-dimensional harmonic oscillator [2]. However much like Janz *et al*., the effect of shear on the liquid was not investigated. Jo *et al*., discarded measurements below 20 s^–1^ due to the limited torque resolution showing the Newtonian behaviour at 300 °C only [3]. Whilst the paper does provide viscosity measurements at 300 °C and 400 °C, it only uses data going back 1979 as a reference. Indeed very little rheology has been carried out on this complex high temperature liquid. Siegel *et al*., also offers viscosity measurements using steady state shear viscosity data through the use of a plate-plate geometry with a 0.1 mm gap offering accuracy of ±10.0% [4]. Finally data digitised from Serrano-López *et al*., does provide numbers with slightly higher values (below 400 °C) than that of all the other published data, however the origin of the measurement as well as the accuracy and the setup are not provided [5].

**Figure 1:**
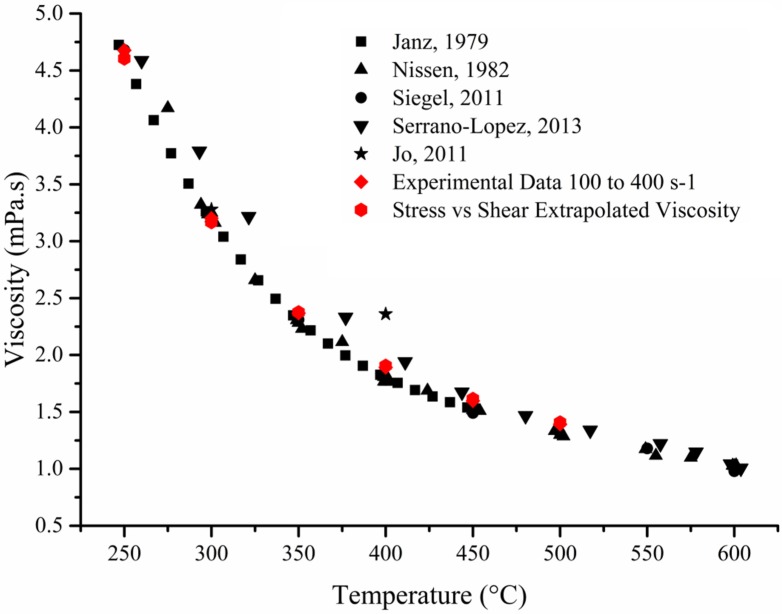
Digitised data taken from various publications [1,2,3,4,5] on the viscosity of eutectic mixture of sodium and potassium nitrate (60/40 ratio) against data acquired on the viscometer and extrapolated from gathered data.

Whilst there is correlation in the data between Janz *et al*., Nissen *et al*., Siegel *et al*., and Serrano-López *et al*., there is large variation in precision and no define values have been agreed upon in the scientific community to ensure accuracy of data (Figure 1) [1,2,4,5]. Furthermore very little evidence of the Newtonian behaviour of salt has been demonstrated at different temperature. This lack of research on basic properties of this mixture is strange as the current growth in renewable energy has led to more research been carried out on molten salt particularly pertaining to the enhancement of specific heat capacity using nano-particles.

Indeed the development and implementation of concentrated solar power (CSP) plant is still faced with technological challenges such as the search for a heat-transfer-fluid (HTF) capable of withstanding higher operating temperature. In many plants, the primary loop systems utilise synthetic oil called VP-1 Therminol (Solutia) [6].

Made up of 26.5% of biphenyl and 73.5% of diphenyl oxide, this fluid displays low operational temperature (390 °C), high vapour pressure, toxicity, as well as high purchasing cost, all of which are pushing industrials and academics to look for alternative materials to solve these challenges [6,7,8]. Using molten salt might be a viable alternative. Indeed binary eutectic mixture of sodium and potassium nitrate (60/40) can withstand temperature of up to 600 °C, display very low vapour pressure are safer to utilize as well as cheaper to purchase. However this material does suffer from lower thermo-physical properties compared to that of synthetic oil as well as higher freezing point placed at 220 °C which imply the use of heat tracing technology. This might be offset by the use of higher operating temperature and therefore higher thermodynamic efficiency [9,10]. Understanding and characterising the viscosity of this mixture would provide a baseline for any other experimental research on how nanoparticles would affect such mixtures.

With HTF playing such an important part in the implementation of this technology, this research demonstrates the Newtonian behaviour of molten salt at both high and low shear rate and analyses previous published and obtained data to produce a correlation of viscosity change against rising temperature which agree well with the different publications. Furthermore, the addition of a low concentration of CuO nanoparticle is investigated and promotes a rise in viscosity as expected.

## 2. Methodology

### 2.1. Sample Production

Sodium nitrate (FISHER, Loughborough, UK) (>99% Pure) and potassium nitrate (SIGMA-ALDRICH, Gillingham, UK) (>99% Pure) were purchased for these tests. The binary mixtures was produced by mixing 12.00 g of NaNO_3_ with 8.00 g of KNO_3_ and melting the blend at 300 °C for 1 h to ensure the homogeneity of the samples. Three samples were tested in the differential scanning calorimeter (DSC) to measure melting point and purity (Table 1) via the Van’t Hoff equations. 

**Table 1:** DSC results of eutectic mixture of sodium and potassium nitrate (60/40 ratio). Purity of the sample was determined to be >99.9%.

60/40 Na/KNO_3_	Weight mg	Integral mJ	Normalized J/g	Onset °C	DSC	Van’t Hoff
Sample 1	36.047	−4047.35	−112.28	221.73	Purity	99.997 ± 3.667 × 10^−3^ mol%
Sample 2	36.357	−4292.85	−118.07	221.73	Impurity	2.917 × 10^−3^ ± 3.667 × 10^−3^ mol%
Sample 3	36.519	−4230.83	−115.85	221.72	T Fusion	222.38 °C

Copper oxide nanoparticle (QinetiQ Nanomaterials Limited, Farnborough, UK) with APS size of 29 nm were dispersed into a binary mixture of nitrate (60 wt % NaNO_3_ + 40 wt % KNO_3_) using a ball-mill (Pascall Engineering, Sussex, UK; BERCO, S/N 17520) and 9 mm stainless steel ball bearing. The resultant mixture containing 0.1 wt % CuO was tested on the viscometer. This equates to a calculated volume fraction of 0.24 or 2,400 ppm given that the density of CuO and the binary mixture is 0.79 g/cm^3^ and 1.899 g/cm^3^ respectively.

### 2.2. Viscometer

An Anton Paar viscometer (Physica MCR 301 TruGap Ready, Anton Paar, St Albans, UK) fitted with a furnace and a temperature controller (TC30) accurate to 0.1 °C was used for these experiments. The geometries employed were a 50 mm diameter stainless steel cone with 1° angle partnered with a 65 mm stainless steel flat plate which was custom made. Once fitted, the motor was warmed up for a period of at least 1 h at 17,000 s^–1^ prior to any measurements been made. This was carried out at 25 °C with a 1.0 mm gap to stabilise the equipment. The motor adjustment was then carried out to calibrate the air bearing for a period of 10 min at 25 °C.

Due to the custom made nature of the bottom plate, it was vital to test few calibration materials to ensure that the whole set up was optimal for the measurements. With a measuring gap locked at 0.098 mm, the volume between the two surfaces is 0.57 cm^3^ which for water at 25 °C equate to a mass of 0.57 g given that its density is 0.997 cm^3^/g. This was used to calculate the amount needed for the different calibration materials used as well as estimate the amount of nitrate eutectic blend required. Providing too much or too little sample would directly affect the results obtained as the area of contact would be altered, it was imperative to use the similar sample size every time as well as ensure the cleanliness of both geometries before and after each measurement.

**Figure 2:**
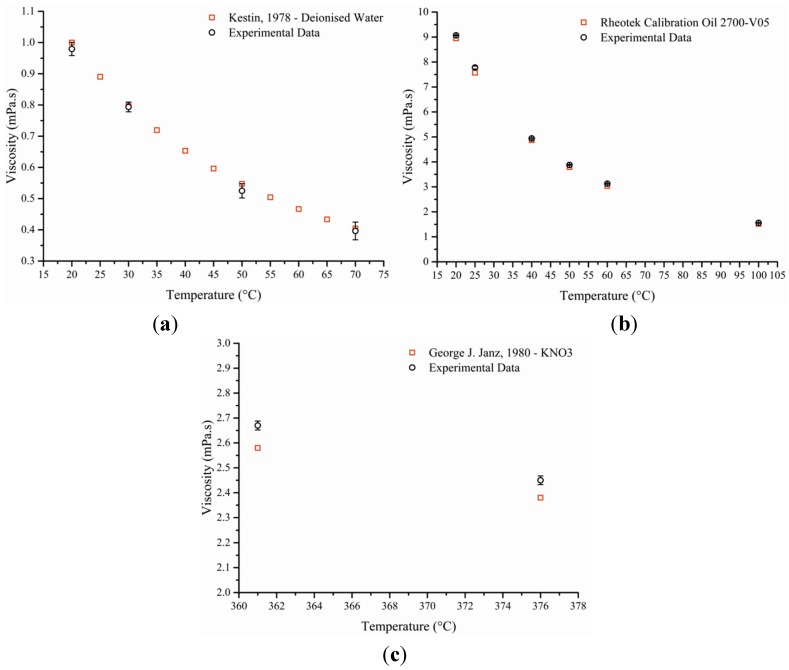
Three graphs showing the difference between experimental data in black to that of published data in red. Water (**a**), calibration oil (**b**) and KNO_3_ (**c**) were used as reference and viscosity was plotted against temperature [1,11,12].

Using water, calibration oil and potassium nitrate, the equipment was tested for precision and accuracy. The calibration carried out on each of these three materials revealed that the instrument was within <4.0% of the reference value (Figure 2) whilst the precision of the results was excellent for both calibration oil and KNO_3_ with values below 1.0% [11,12,13]. Testing deionised water was trickier at higher temperature (50 °C and 70 °C) due to its high vapour pressure leading to evaporation which in turn would alter contact area with the upper plate altering the results (lower precision). This could only be solved through a rapid set up of the tests which were carried out once for each samples and repeated numerously. This was not the case with the other two compounds which had low vapour pressures.

Setting up the viscometer for calibration with oil and water was a straight forward procedure as both materials are liquid at 25 °C and the temperatures used are low and therefore safe to handle. However potassium nitrate is solid at room temperature and only starts to melt at 334 °C. Therefore the amount of solid needed had to be calculated from the density which decreased linearly as measured by Janz *et al*., with values varying from 1.847 g/cm^3^ at 361.85 °C to 1.836 g/cm^3^ at 376.85 °C [13]. Using a value of 1.85 g/cm^3^, ≈1.055 g of KNO_3_ was placed in the centre of the bottom plate. The cone was lowered so as to allow the closure of the furnace and set 1 or 2 mm above the sample. Then the temperature was brought up to 361 °C. Once it had stabilised, the cone was moved into its measuring position of 0.098 mm and the experiment was started. This same method was applied for the eutectic mixture of sodium and potassium nitrate (60/40 wt % respectively, 1.899 g/cm^3^) except that the starting temperature was set at 250 °C (8 samples were tested at each temperature) and that the amount used was slightly higher at 1.08 g. This was repeated for the copper oxide tests. The tests for oil, water and potassium nitrate were carried out at a constant shear rate of 200 s^–1^ (Figure 2 and Table 2). 

The eutectic mixture of nitrate was tested at both high and low shear rate with the following setting:

- 100 s^–1^ to 400 s^–1^—Linear increase in shear—Measuring point duration 120 s;
- 100 s^–1^ to 1 s^–1^—Logarithmic decrease in shear—10 points per decades—Measuring point duration 120 s;

The molten salt mixture doped with 0.1 wt % CuO was tested using the following setting:

- 100 s^–1^ to 1000 s^–1^ with 10 points per decades.

**Table 2:** Percentage difference between the average measurement and the referenced value.

**Temperature in Degree Celsius**	**Deionised Water**
20	−2.063
30	−0.483
50	−4.049
70	−1.854
**Temperature in Degree Celsius**	**Oil Standard**
20	1.341
25	2.774
40	1.437
50	2.375
60	2.961
100	2.632
**Temperature in Degree Celsius**	**KNO_3_**
361	3.488
376	2.941

## 3. Results and Discussion

**Figure 3:**
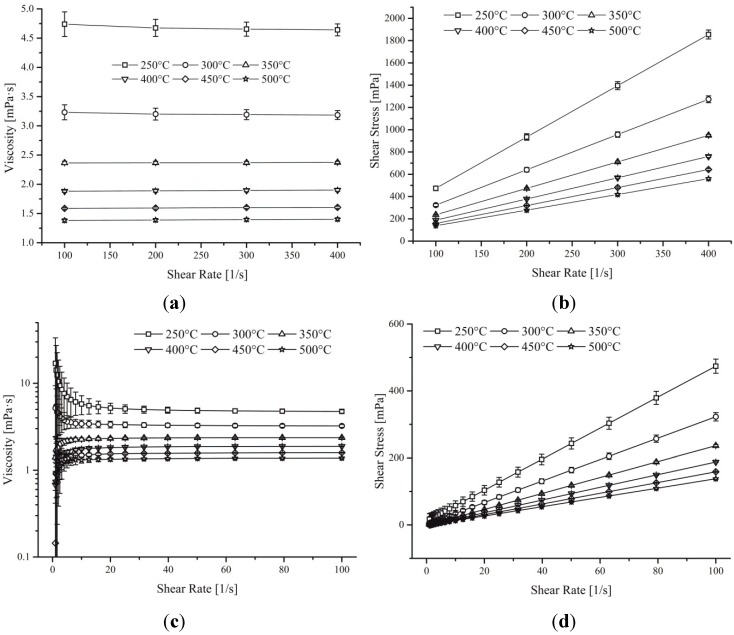
(**a**) and (**b**) graphs show the effect of viscosity and shear stress against changes in shear rate (100 s^–1^ to 400 s^–1^) for different temperatures. (**c**) and (**d**) graphs display the effect of viscosity and shear stress against decreasing shear rate from 100 s^–1^ to 1 s^–1^ (Value below 20 s^–1^ were discarded due to low torque resolution).

Viscosity data showed in Figure 3a no doubt as to the Newtonian behaviour of this eutectic mix of sodium and potassium nitrate. Indeed the constant viscosity with varying shear rate as well as the linear gradient of stress *vs*. shear are testimony of the behaviour of this high temperature fluid.

**Figure 4:**
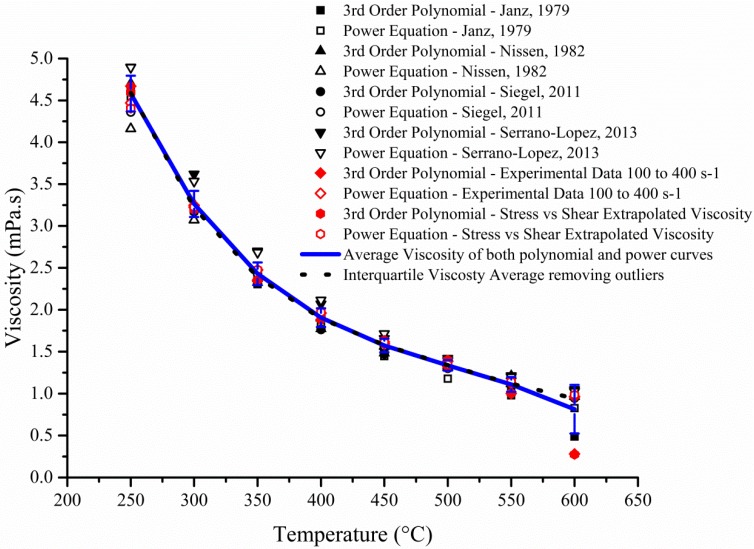
3rd Order polynomial and power trend line fitted for each of the digitised data plotted on Figure 1 as well as the experimental data for 100 s^–1^ to 400 s^–1^ and the extrapolated viscosity from the Stress *vs*. Shear graph (gradient of each line on Figure 3b).

However, the accuracy of the data is something that is still open to debate as very little rheology has been accumulated on these complex high temperature fluids. Different publications have suggested various correlations to fit their respective values leading to the production of many equations, all of which vary slightly from one to the next [5]. To solve this problem, both the published and experimental data were fitted with third order polynomial as well as power line of best fit (Figure 4). The 12 sets of data were averaged in two different ways where one took into account all the data even outliers whilst the other removed the latter from the calculation (Dotted black line on Figure 4). The interquartile average was used to fit a 4th order polynomial (1) and a power trend-line (2) producing the following equations:

*y* = 3.69483 × *10^–10^* × *x^4^* – 7.63950 × *10^–7^* × *x^3^* + 5.96378 × *10^–4^* × *x^2^* – 2.21682 × *10^–1^* × *x* + 3.0983 × 10 (1)

*y* = 79786 × *x^–1.773^* (2)

The fitting of the 4th order polynomial gave an *R^2^* value of 9.99997 × 10^–1^ whilst the power trend-line was fitted with a value of 0.9986. Both equations were then employed to test its accuracy against the collect digitised data to analyse the absolute percentage variation.

**Figure 5:**
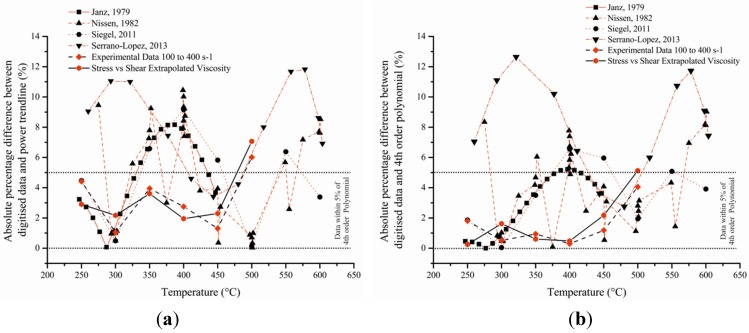
Absolute percentage of digitised data against power trend line (**a**—Graph) and 4th order polynomial (**b**—Graph) where the temperatures of the obtained data as well as experimental and extrapolated (Figure 3b—Line Gradient) numbers were used to calculate the theoretical values with the two different equations.

**Figure 6:**
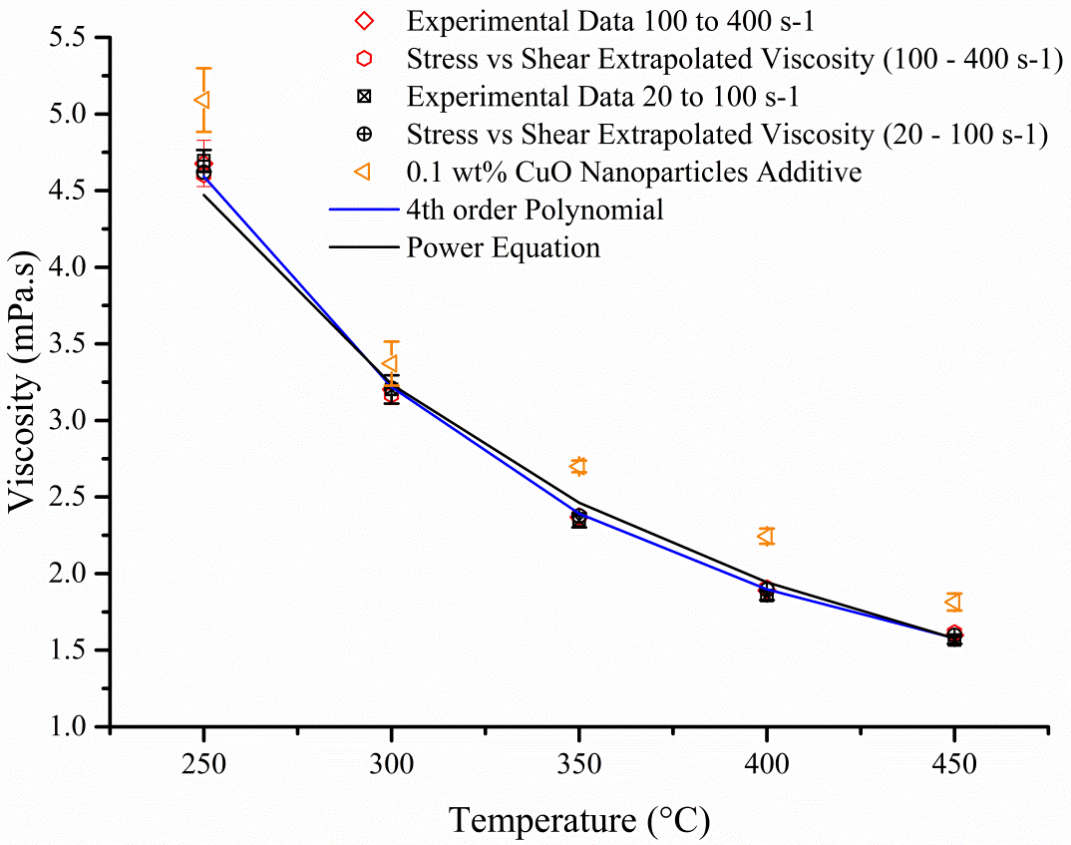
Viscosity of molten salt against temperature. Addition of 0.1 wt % CuO led to an increase of 10.8%, 4.7%, 12.9%, 18.3%, 14.9% for 250 °C, 300 °C, 350 °C, 400 °C, 450 °C respectively when compared to the 4th order polynomial.

Whilst more variations were seen with the power trend line fitting (Figure 5a), the absolute percentage difference showed that the 4^th^ order polynomial (Figure 5b) fitted most of the data within a 5.0% error margin. This was also showed in Figure 6 where the 4th order polynomial and the experimental data fitted accurately. As expected (Figure 6), the addition of nanoparticles to the mix led to an increase in viscosity which is probably due to the formation of vortex structure near the surface of the particle promoting a rotational movement [14]. This extra expenditure of energy will promote an increase in viscosity which will also be affected by the shape (sphere, plate or rods) of the nanoparticles where aspheric particles are responsible for higher viscosity and their spherical counterpart [15]. Furthermore the shear rate will affect particle-particle interaction and the latter should grow weaker with rising velocity [16]. Other parameters such as loading factor, type of nanoparticles, size and fluids utilize will drastically impact the viscosity.

The viscosity of molten salt is going to affect the cost of pumping this liquid through the CSP pipes and knowing its behaviour is critical to the development and use of this type of HTF. The addition of any type of nanoparticles is likely to promote an increase in viscosity.

## 4. Conclusions

**Figure 7:**
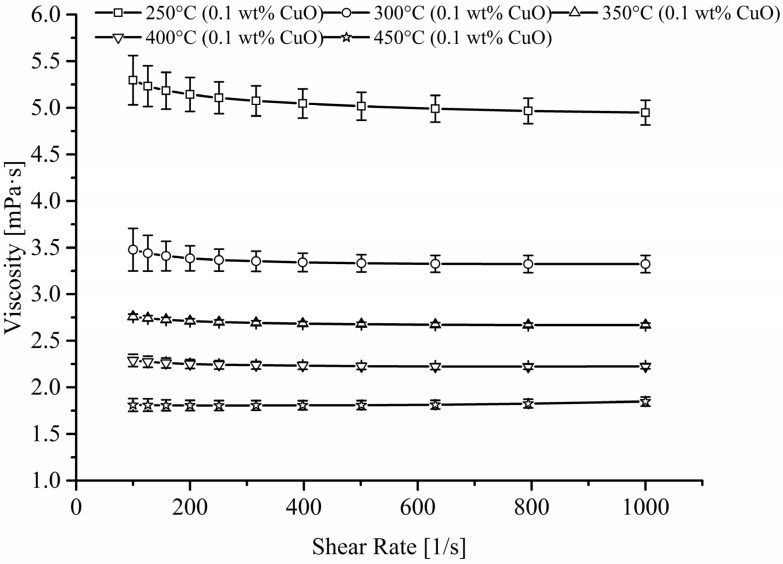
Effect of viscosity against rising shear rate when 0.1 wt % CuO nanoparticles is added to a binary eutectic mixture of sodium and potassium nitrate.

The paper clearly demonstrates the Newtonian behaviour of this complex mixture whether that is at high or low shear rate although torque resolution was no high enough below 20 s^–1^. Analysis of the pooled data produced a 4th order polynomial equation which fitted published and obtained data accurately. The addition of very low level of nanoparticles led to a rise in viscosity as anticipated and no shear thinning effect was observed (Figure 7).

The use of molten salt in CSP as an HTF could allow the plant to run at higher temperature with therefore greater efficiency. This might not necessarily reduce the levelised cost of electricity even if storage is used as heat tracing technology needs to be implement which is a costly process to put in place. Whilst the low vapour pressure and viscosity reduces the cost of pumping the HTF through the plant, its low specific heat capacity and the current research on the addition of nanoparticles is likely to raise it as showed in this paper.
